# Seasonal abundance of *Anopheles* mosquitoes and their association with meteorological factors and malaria incidence in Bangladesh

**DOI:** 10.1186/1756-3305-7-442

**Published:** 2014-09-18

**Authors:** Kabirul Bashar, Nobuko Tuno

**Affiliations:** Laboratory of Entomology, Department of Zoology, Jahangirnagar University, Savar, Dhaka 1342 Bangladesh; Laboratory of Ecology, Faculty of Natural Science and Technology, Kanazawa University, Kanazawa, Japan

## Abstract

**Background:**

The relationship between climatic factors and mosquito abundance is very important to determine parasite activity levels and, therefore, disease risk. Therefore, this study was conducted to investigate the seasonal abundance of anophelines and their association with meteorological variables and disease transmission in two malaria endemic areas of Bangladesh.

**Methods:**

Monthly sampling was done from both indoors and outdoors in 12 selected houses using light traps (LTs) and pyrethrum spray (PS) during January, 2011 to January, 2012 in two malaria endemic areas of Bangladesh. Outdoor rainfall, temperature, and relative humidity data of the study areas were collected from the meteorological department of Bangladesh. Mosquitoes were killed with chloroform and identified morphologically under stereoscopic microscopes using taxonomic keys. Samples were tested for CSP of *P. falciparum*, *P. vivax* 210 and *P. vivax* 247 using ELISA. Pearson correlation and canonical correspondence analyses (CCA) were computed to investigate the associations with species abundance and rainfall, temperature, humidity and malaria cases.

**Results:**

A total of 2,443 female anophelines, representing 22 species were captured. Every female *Anopheles* were tested for *P. falciparum*, *P. vivax* 210 and *P. vivax* 247 CSP, of which 10 species were found positive. The CSP positive species were *An. annularis*, *An. baimaii*, *An. barbirostris*, *An. jeyporiensis*, *An. karwari*, *An. minimus* s.l.*, An. philippinensis*, *An. umbrosus*, *An. vagus* and *An. wilmori. Anopheles vagus* and *An. philippinensis* were the dominant species present almost throughout the year with highest peaks in March and smallest peaks in September but *An. baimaii* and *An. willmori* were found during monsoon (July -September) only. Lag rainfall and relative humidity were the most significant variables influencing *An. baimaii*, *An. willmori*, *An. vagus*, and *An. subpictus* density in Kumari area. Abundance of these four species positively related to malaria cases. The effects of temperature were not found as a significant variable on the abundance of anophelines mosquitoes in Bangladesh.

**Conclusions:**

Our study demonstrates that the nature of relationship between malaria vector and climatic variables were multifaceted. Detailed studies of vector bionomics, continuous monitoring and malaria transmission dynamics is essential for predicting disease outbreaks and vector control in the region.

## Background

Malaria is one of the most formidable and serious public health problems in Bangladesh [1]. It is endemic in 13 northern and eastern areas bordering India and Myanmar, with 90% of morbidity and mortality reported from Rangamati, Bandarban and Khagrachari districts [2]. The malaria situation in Bangladesh is complex due to high species diversity and species complexes with many sibling species presenting different ecological behaviors [2–7]. *Anopheles baimaii* Sallum and Peyton, 2005 *(dirus D), An. minimus* s.l. Theobald, 2001, *An. philippinensis* Ludlow*,* 1902*,* and *An. sundaicus* (Rodenwaldt, 1925) are considered as primary and *An. aconitus* Doenitz*,* 1902, *An. annularis* Van der Wulp, 1884, and *An. vagus* Doenitz, 1902 as epidemic malaria vectors in Bangladesh [4]. However, recent studies [1, 8, 9] have reported *An. nigerrimus* Giles, 1900, *An. subpictus* Grassi*,* 1899, *An. barbirostris* Van der Wulp, 1884, *An. maculates* Theobald, 1901, *An. jeyporiensis* James*,*1902, *An. karwari* James, 1902, *An. kochi* Doenitz*,*1901, *and An. peditaeniatus* (Leicester, 1908) as *Plasmodium* positive.

Environmental changes have a great bearing on breeding habitats of different mosquito species that control adult population density [10]. Meteorological factors affect adult mosquito abundance by altering the quality and quantity of breeding habitats. The relationship between climate variables and mosquito abundance can provide important information to determine parasite activity levels and, therefore, disease risk [11–16]. Exact information on the seasonal prevalence of mosquito fauna in a region is essential for the development of efficient vector control programs [17]. But there were few published data on the seasonal abundance of anopheline mosquitoes in Bangladesh. Therefore, this study was conducted to investigate the species composition and population dynamics of anophelines and their association with meteorological variables and disease transmission in two malaria endemic areas of Bangladesh.

## Methods

### Study areas

**Figure 1:**
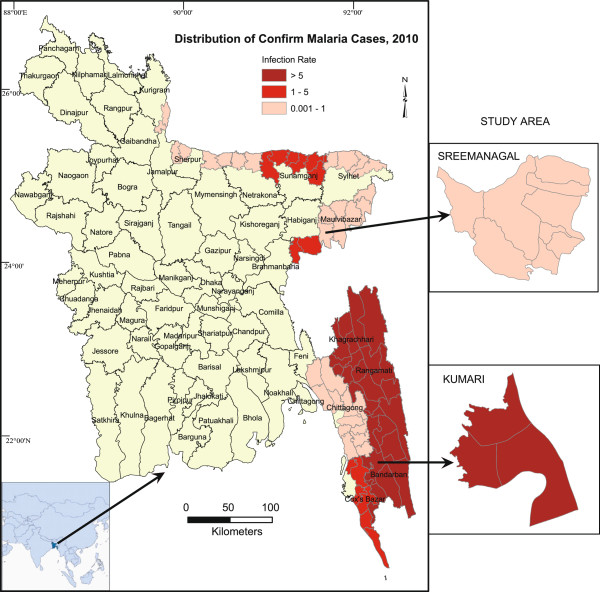
**Study areas and distribution of malaria cases in Bangladesh.**

Two malaria endemic areas of Bangladesh were selected for the study these are Kumari of Bandarban district (21° 44' N, 92° 8'E) and Sreemangal of Maulvibazar district (24° 19' N, 91° 46' E) (Figure 1). These areas were selected on the basis of the malaria prevalence and bio- geographical condition of Bangladesh. Infection rate in Kumari and Sreemangal was 5 > and 0.001-1 respectively [8]. A major part of Kumari is vegetated by secondary forest with interspersed rubber plantations. Hot and humid climatic conditions, narrow slow-running streams, wells, pools and rice fields in the area are the suitable breeding habitats for mosquitoes. Semi-evergreen coniferous forests are the major vegetation in Sreemangal. There are few slow running streams which acted as mosquito breeding sources. Ggeographical positions of the sampling places were recorded using handheld GPS (Garmin Oregon 550). ArcView GIS 3.3 and Arc GIS 9.2 software were used to map the sampling area.

### Mosquito collections and identification

Mosquitoes sampling was done twice in a month during January to December’ 2011 in Kumari and once in a month during February to December’ 2011 and January’ 2012 in Sreemangal. Mosquitoes were captured from both indoors and outdoors from 12 selected houses from the study areas using light traps (LTs) and pyrethrum spray (PS) following World Health Organization procedures [18]. Collected mosquitoes were brought to the field laboratory for processing and identification. Mosquitoes were killed with chloroform and identified morphologically under stereoscopic microscopes within 12 hour using taxonomic keys [19–22] and stored in Eppendorf tubes with soft tissue paper and silica gel desiccant for stable storage at room temperature for further process. Collected mosquitoes were transported to the laboratory at Jahangirnagar University to be preserved in freezer. The anophelines were brought to Kanazawa University later to confirm species identification.

### Sample preparation and CSP ELISA

Each mosquito was bisected into head–thorax and abdomen. The anterior part, head, and thorax of the mosquitoes were tested. Samples were tested for CSP of *Plasmodium falciparum* (Pf), *P. vivax* (Pv) 210 and *P. vivax* 247 using ELISA as described by Burkot et al. [23] and Wirtz et al. [24] with slight modification. All monoclonal antibodies were collected from the Centers for Disease Control and Prevention (CDC), Atlanta. These were used to detect and distinguish the circumsporozoite proteins (CSP) of *P. falciparum* and two distinct polymorphs of *P. vivax*: Pv-210 and Pv-247. Four negative controls (each the triturated whole body of a laboratory reared, *Aedes aegypti*) and one positive control (supplied by CDC) were included in each microtitre plate. ELISA results (i.e. change in color) were visually judged according to the color intensity (no color change, negative result), comparable with the positive control. All positive samples were re-tested for confirmation. We considered the sample as CSP-positive, if two of two trials were positive. Recently Durnez et al. [25] and Bashar et al. [9] describe a new methodology to check the true CSP positive. They proved that, ELISA reacting antigen of *Plasmodium* was heat-stable but false positive causal agent was not. Therefore we confirm positive results by a second CSP-ELISA test applying heated the samples at 100°C for 10 minutes.

### Ethics statement

Ethical clearance was obtained from the head of each village, office of the local government and the ethics committee of Bangladesh Medical Research Council (No. PU/203/11). We took verbal consent from all of the household head for this study because the majority of them are illiterate. The ethics committee approved this consent procedure.

### Malaria cases

Data on the malaria cases (*Pf* and *Pv*) of the study areas were received from the office of malaria control program and local Hospitals. Local hospitals routinely checked the *P. falsiparum* cases using rapid diagnostic test (RDT) and *P. vivax* cases by using thin and thick blood films.

### Meteorological data

Outdoor rainfall, temperature and relative humidity data of the study areas, from January’ 2011 to February’ 2012 were collected from the local stations of the meteorological department of Bangladesh. Study areas are located in a tropical monsoon climate characterized by marked seasonal variations. Abundant rainfall (monthly average 595.12 mm) during the monsoon (July-October) is followed by a cool winter period (November-February), then a hot, dry summer (March-June). The winter season is dry and accounts for 2- 4% (average 1.88 mm) of the annual rain. As the winter season progress into the summer hot season, rainfall increase (10-35% of the total annual rain). In the hot summer season, the average maximum temperature is 34°C and the minimum is 21°C. Average maximum temperature in winter is 29°C and the minimum is 11°C. The average monthly relative humidity for the whole year ranges from 63.13 to 87.73%. The relative humidity is over 80% during June to September. March and April are the least humid months.

### Data analysis

In order to compare counts of female anophelines captured in different periods, we determined the relative density of every mosquito species according to the formula of Kocatas [26] and Simsek [27], in which RD (relative density) = NA (number of all specimens of each species collected during each period)/N (the number of specimens of all species collected during each period) × 100. Statistical analyses were done on abundance data of the predominant species captured during the study to find out which environmental variables were leading on species distributions in the study areas. Pearson correlation coefficients were computed for the dominant species using SPSS® 16.0 (© SPSS Inc., Chicago, IL. 2007) to study the correlations between mosquito abundance and environmental variables. It was also calculated to know relation between malaria morbidity and environmental variables. Pearson correlation analysis between malaria morbidity and environmental variables were not done with the data of Sreemangal area because morbidity was not recorded in this area during the study period. Canonical correspondence analyses (CCA) [28] have been done using CANOCO 4 for Windows [29] to further explore the associations between species abundance with meteorological variables. It is a multivariate direct gradient analysis technique, where species abundance and composition is directly related to a set of meteorological variables. CCA was carried out in our study because meteorological variables may be highly correlated with one another. For example, rainfall, temperature and humidity are usually very tightly correlated. If so, any one of these variables could be used as a proxy for all others. Generally, it is best to choose the variable which is most likely to be the direct cause of species response, and/or a variable which has been used in other ecological studies. It might not be known beforehand which variables are correlated with each other. In this case, a detailed examination of the correlation matrix would be helpful. A relatively sophisticated way to do this would be to perform CCA. Moreover, it performs well with skewed species distributions, high noise levels, and complex sampling designs [30]. Traditional analyses were used to calculate the minimum infection rate (MIR) of CSP and to estimate the population infection rate from pooled samples. When a pool was positive, only one individual in that pool was considered to be positive.

## Results

### Species composition and density

A total of 2,443 female anophelines, representing 22 species were collected in two selected areas during January 2011 to February 2012 (Table 1). Among the 22 species collected, the most prevalent species was *An. vagus* (n = 1263, 51.70%) followed by *An. philippinensis* (n = 560, 22.92%), *An. jeyporiensis* (n = 143, 5.85%), *An. karwari* (n = 108, 4.42%) and *An. peditaeniatus* (n = 97, 3.97%) respectively. Few number of samples (n < 10) was collected in the following 10 species; *An. aitkenii*, *An. baimaii, An. nigerrimus, An. nivipes*, *An. pallidus*, *An. pseudojamesi*, *An. tessellatus, An. maculatus*, *An. hyrcanus* group, and *An. willmori* (Table 1).

**Table 1:** **Female anophelines collected in indoor and outdoor using light traps and pyrethrum spry (PS) from Kumari (Jan-Dec’11), Bandarban and Sreemangal (Feb-Dec’11 & Jan’12), Moulvibazar, Bangladesh**

Species	Kumari					Sreemangal			Grand Total	
	Light trap			PS	Total	Light trap		Total	N	RD ^1^
	Indoor	Outdoor	Total	Indoor		Indoor	Outdoor			
*Anopheles aitkenii*	1		1		1				1	0.04
*An. annularis*	3	7	10	1	11	3	1	4	15	0.61
*An. baimaii*	2	1	3		3				3	0.12
*An. barbirostris*	7	3	10		10				10	0.41
*An. hyrcanus* group	4	2	6		6				6	0.25
*An. jamesii*	35	20	55	2	57				57	2.33
*An. jeyporiensis*	62	71	133	5	138	3	2	5	143	5.85
*An. karwari*	30	76	106		106	2		2	108	4.42
*An. kochi*	16	17	33		33				33	1.35
*An. maculatus* group	2	4	6		6				6	0.25
*An. minimus* s.l.	26	19	45	1	46				46	1.88
*An. nigerrimus*	2	1	3		3				3	0.12
*An. nivipes*	2	2	4		4				4	0.16
*An. pallidus*	4		4		4				4	0.16
*An. peditaeniatus*	24	57	81	1	82	1	14	15	97	3.97
*An. pseudojamesi*	3	1	4		4				4	0.16
*An. philippinensis*	268	289	557	2	559		1	1	560	22.92
*An. subpictus*	14	36	50	1	51				51	2.09
*An. tessellatus*	1	4	5		5				5	0.20
*An. umbrossus*	9	7	16		16				16	0.65
*An. vagus*	643	454	1097	165	1262	1		1	1263	51.70
*An. willmori*	5	3	8		8				8	0.33
Grand Total	1163	1074	2237	178	2415	10	18	28	2443	100.00

^1^Relative density.

### Species composition in Kumari

In total, 2415 female *Anopheles* mosquito belonging to 22 species were captured in Kumari using LTs (n = 2237) and PS (n = 178). All 22 species were collected using LTs set indoors (n = 1163), and 20 species were collected with outdoor LTs (n = 1074). In contrast, only eight species were collected using PS (n = 178). *Anopheles vagus* was captured dominant in both LTs and PS in indoor. Relatively high number of *An. karwari* (n = 76) and *An. peditaeniatus* (n = 57) were captured in outdoor (Table 1).

### Species composition in Sreemangal

Small number (n = 28) of female anophelines were captured using light trap set both indoors and outdoors during one year sampling period in Sreemangal. Only six species; *An. peditaeniatus*, *An. jeyporiensis*, *An. annularis*, *An. karwari*, *An. philippinensis*, and *An. vagus* were collected from this area. *Anopheles peditaeniatus* (n = 15) was the dominant species followed by *An. jeyporiensis*, and *An. annularis. Anopheles vagus* and *An. karwari* were captured only in indoors (Table 1).

### Gonotrophic stages

Gonotrophic stages of mosquito were visually classified as unfed (UF), Blood-fed (BF), semi-gravid (SG), or gravid (G). The highest percentage of specimens was UF (n = 1601, 65.53%), followed by BF (n = 697, 28.53%), G (n = 91, 3.72%), and SG (n = 54, 2.21%).

### Circumsporozoite positive (CSP) rates

Every female *Anopheles* were tested for *P. falciparum*, *P. vivax* 210 and *P. vivax* 247 CSP, of which 10 species were found positive. The CSP positive species were *An. annularis*, *An. baimaii*, *An. barbirostris* Van der Wulp, *An. jeyporiensis*, *An. karwari*, *An. minimus* s.l.*, An. philippinensis*, *An. umbrosus*, *An. vagus* and *An. wilmori.* A total of 22 (0.91%) mosquitoes belonging to eight species were found positive for *P. falciparum*, 10 (0.41%) mosquitoes belonging to five species were positive for *Pv*-210 and one (0.04%) mosquitoes belonging to one species were positive for *Pv*-247. Mixed infections were found in 5 females (0.20%) anophelines. In total of *P. falciparum* and *P. vivax* infections, the highest infection rate was observed in *An. baimaii* (2/3, 66.66%) followed by *An. wilmori* (2/8, 25%), *An. barbirostris* (1/10, 10%), *An. annularis* (1/13, 7.69%), *An. umbrosus* (1/16, 6.25), *An. karwari* (3/132, 2.27%), *An. vagus* (21/1251, 1.68%), *An. minimus* s.l. (1/64, 1.56%), *An. jeyporiensis* (1/98, 1.02%), and *An. philippinensis* (5/554, 0.90%) (Table 2). No *Anopheles* was found CSP positive*,* collected from Sreemangal, probably because of the low sample size (n = 28).

**Table 2:** **Species and number of** ***Anopheles*** **tested and their CSP positive rates**

***Anopheles***species	Number tested	CSP positive					Infection rate (%)
		Pf	Pv210	Pv247	Mixed (Pf, Pv210)	Total	
*aitkenii*	2	-	-	-	-	0	0.00
*annularis*	13	1	-	-	-	1	7.69
*baimaii*	3	2	-	-	-	2	66.67
*barbirostris*	10	1	-	-	-	1	10.00
*hyrcanus*	6	-	-	-	-	0	0.00
*jamesii*	57	-	-	-	-	0	0.00
*jeyporiensis*	98	-	-	1	-	1	1.02
*karwari*	132	1	2	-	-	3	2.27
*kochi*	36	-	-	-	-	0	0.00
*maculatus*	7	-	-	-	-	0	0.00
*minimus* s.l.	64	-	1	-	-	1	1.56
*nigerrimus*	3	-	-	-	-	0	0.00
*nivipes*	4	-	-	-	-	0	0.00
*pallidus*	8	-	-	-	-	0	0.00
*peditaeniatus*	82	-	-	-	-	0	0.00
*pheudojamesi*	4	-	-	-	-	0	0.00
*philippinensis*	554	1	3	-	1	5	0.90
*splendidus*	1	-	-	-	-	0	0.00
*subpictus*	51	-	-	-	-	0	0.00
*tessellatus*	5	-	-	-	-	0	0.00
*umbrosus*	16	1	-	-	-	1	6.25
*vagus*	1251	14	3	-	4	21	1.68
*willmori*	8	1	1	-	-	2	25.00
**Total**	**2415**	**22**	**10**	**1**	**5**	38	1.57

### Seasonal prevalence in Kumari

**Figure 2:**
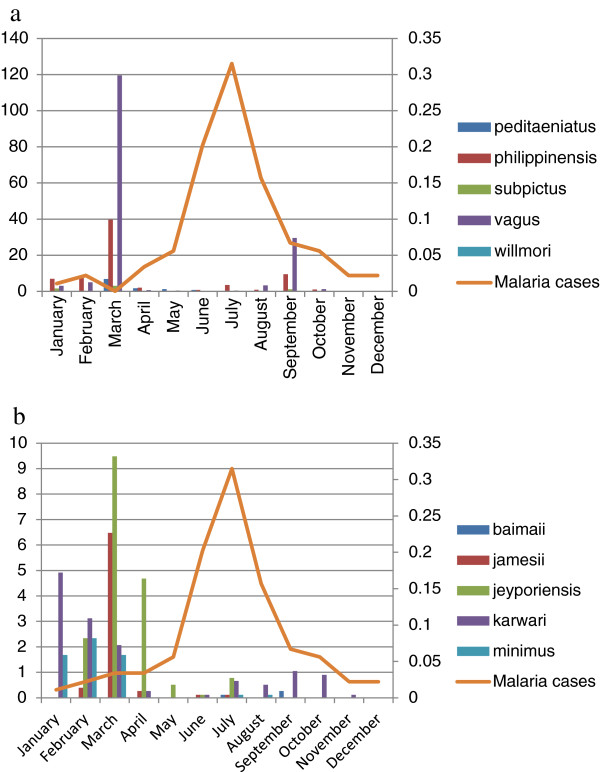
**Monthly abundance of anophelines species and malaria morbidity in Kumari. a**. *Anopheles peditaeniatus, An. philippinensis, An. subpictus, An. vagus, An. willmori* and malaria cases. **b**. *Anopheles baimaii, An. jamesii, An. jeyporiensis, An. karwari, An. minimus* and malaria cases.

The seasonal distributions of the most abundant species reveal population fluctuations in different months. *Anopheles vagus* and *An. philippinensis* were the dominant species present almost throughout the year with major peaks in March and smaller peaks in September but *An. baimaii* and *An. willmori* were found during monsoon (July -September) only. Although the population of *An. karwari* decreased greatly in May and December, it was collected in small numbers throughout the sampling period with peaks in February. Highest and lowest densities of anophelines were found in March and December respectively in Kumari (Figure 2).

### Seasonal prevalence in Sreemangal

Mosquito density was reached peak during April in Sreemangal. The density of *An. minimus* s.l. was higher during January to March. Abundance of *An. annularis* followed the same general trend as that for *An. minimus* s.l. but with smaller numbers.

### Association with climatic variables and morbidity

**Figure 3:**
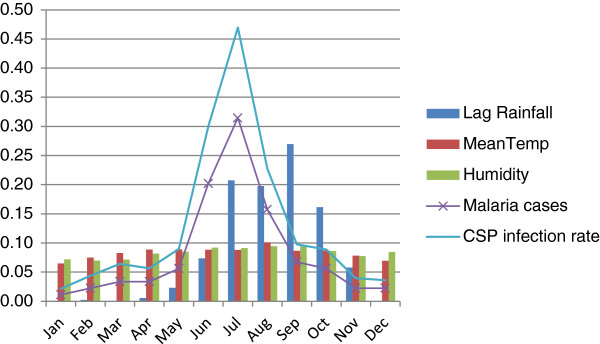
**Correlation among CSP infection, malaria morbidity and environmental variables in Kumari.**

Bivariate Pearson’s correlation analyses of most abundant species with climatic variables showed that the abundance of *An. baimaii* (*p <* 0.01) and *An. willmori* (*p <* 0.01) has strong positive correlation with lag rainfall in Kumari. But we did not find any significant relation with rainfall and most other *Anopheles* species in Kumari as well as in Sreemangal. The effect of temperature were not found as an important variable on the abundance of anophelines mosquitoes in both the study areas except *An. karwari* (R = −0.58, *p <* 0.05). Strong negative correlation between relative humidity and abundance of *An. karwari* (*p <* 0.01), *An. minimus* s.l. (*p <* 0.01), *An. annularis* (*p <* 0.01), and *An. jeyporiensis* (*p <* 0.01) were observed in our study. We found significant relation between *An. willmori* (*p <* 0.05) and malaria morbidity when Spearman’s correlation analyses was done. Insignificant impact of 1 month lag rainfall and temperature on the malaria morbidity were found in Kumari. Highest number of malaria cases was recorded during July but rainfall was higher during August (Figure 3). Significant positive correlation were observed between malaria cases and relative humidity (*p <* 0.05) in Kumari (Table 3).

**Table 3:** **Pearson’s correlation coofficient (R) of most abundance species with climatic variables and their significant (** ***p*** **) value**

Place	Species	Lag Rain fall		Mean Temperature		Relative Humidity		Malaria morbidity	
		R	***p***-value	R	***p***-value	R	***p***-value	R	***p***-value
Kumari	*An. baimaii*	0.737	0.006^*^	0.175	0.587	0.485	0.110	0.312	0.323
	*An. jeyporiensis*	−0.409	0.187	0.26	0.935	−0.503	0.096	−0.224	0.484
	*An. karwari*	−0.275	0.388	−0.582	0.047^*^	−0.664	0.019^*^	−0.325	0.303
	*An. minimus* s.l.	−0.467	0.126	−0.511	0.089	−0.804	0.002^*^	−0.347	0.270
	*An. peditaeniatus*	−0.368	0.239	0.078	0.809	−0.383	0.219	−0.181	0.574
	*An. philippinensis*	−0.192	0.549	−0.107	0.740	−0.466	0.127	−0.199	0.535
	*An. vagus*	−0.125	0.700	0.005	0.988	−0.338	0.282	−0.188	0.559
	*An. willmori*	0.957	0.00^*^	0.510	0.09	0.694	0.012^*^	0.486	0.110
Sreemangal	*An. annularis*	−0.332	0.292	−0.156	0.628	−0.734	0.007^*^	-	-
	*An. jeyporiensis*	−0.295	0.352	0.082	0.801	−0.747	0.005^*^	-	-
	*An. peditaeniatus*	−0.171	0.594	0.127	0.694	−0.446	0.146	-	-

**Figure 4:**
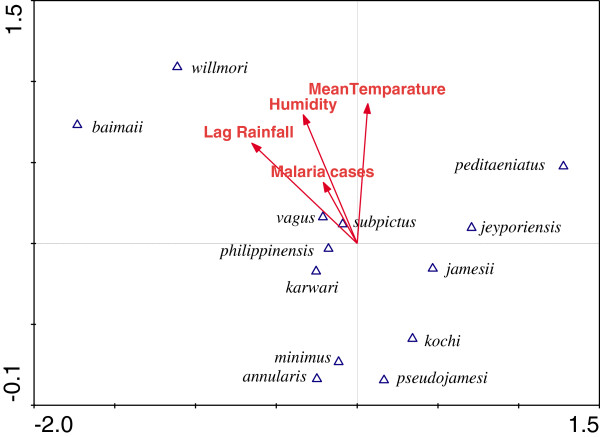
**Ordination diagram of most abundance anophelines species and environmental variables of Kumari, Bandarban, produced from canonical correspondence analysis (CCA).**

**Figure 5:**
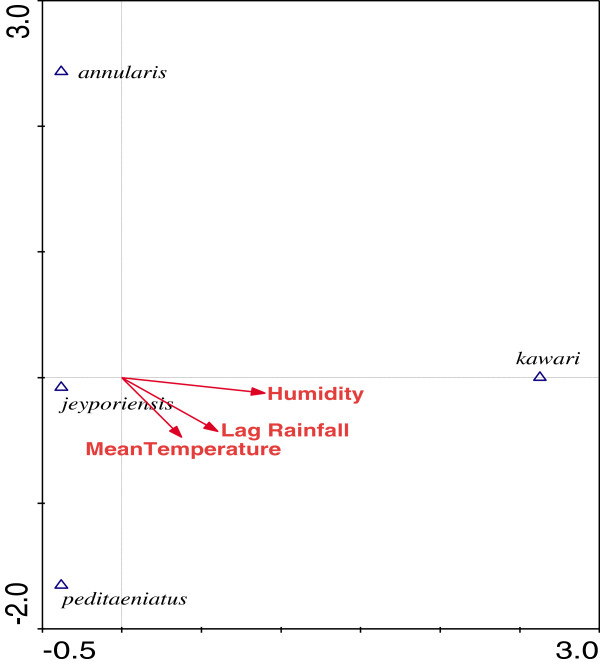
**Ordination diagram of most abundance anophelines species and environmental variables of Sreemangal, produced from canonical correspondence analysis (CCA).**

CCA was performed to further explore the associations between species abundance with meteorological variables. The ordination diagram produced by CCA (Figure 4 & 5) shows the relationships between species abundance and meteorological variables in Kumari and Sreemangal. The triangles represent species abundance and the arrows are meteorological variables. The direction and length of the arrow indicates the association of the variable and how it correlates with the species composition axes. The angle between arrows shows correlations among the climatic variables. The position of the triangles (species) exposes the environmental preferences of each species. Lag rainfall and relative humidity were the most significant variables influencing *An. baimaii*, *An. willmori*, *An. vagus*, and *An. subpictus* density in Kumari. Abundance of these four species was positively related to malaria cases. The abundance of other species was negatively associated with environmental variables (Figure 4). We did not find any positive association with environmental variables and female anophelines abundance in Sreemangal (Figure 5). Canonical Correspondence Analysis (CCA) showed a low species-environment correlation with Eigenvalues (λ) of the first, second, third and fourth axes of 0.195, 0.107, 0.044, and 0.033, respectively. The Eigenvalue is a reasonable measure of the strength of an ordination axis. It is actually equal to the (maximized) dispersion of the species scores on the ordination axis, and is thus a measure of importance of the ordination axis. The first ordination axis has the largest Eigenvalue, the second axis is the second largest Eigenvalue, and so on. The Eigenvalues of CCA all lie between 0 and 1. Values over 0.5 often denote a good separation of the species along axis.

## Discussion

Highest peaks of the female anophelines species density were observed during March and smallest peaks during September in Kumari and there were no positive relation with rainfall, and majority of the anopheline species. We did not found any significant correlation with rain fall, and known malaria vector; *An. minimus* s.l., *An. philippinensis*, *An. vagus*, and *An. annularis*. It may because of association with rice fields and irrigated cropland, where the females deposit their eggs on moist soil, which was supported by Meisch [31]. Investigation is needed in these respects in Bangladesh. Like us, Rahman *et al.* [32] did not found correlation between density and rainfall in Malaysia. The observed results may be because Bangladesh has very high vector species diversity and vectors suited to different breeding habitats. There was mark reduction of mosquito abundance during May to August which increased back in September in our study. The species density reduction during this period may be due to the heavy rainfall, which flushes out breeding sites, larvae, and pupae. It also causes mechanical damage and egg mortality, therefore, reduces the adult abundance [33]. Usually, mosquitoes get optimum (22° - 30°C) temperature [34] for rising population after the winter (March and April) in Kumari. The abundance of principle malaria vector; *An. baimaii* and suspected vector; *An. willmori* were found strongly associated with rainfall in our study. *Anopheles baimaii* inhabit forested mountains and foothills, cultivated forests, plantations (e.g. rubber) and forest fringes [35] and *An. willmori* breeds in slow running stream margins, rice fields, pits and wells [36], seems to be positively associated with rainfall [37]. Olson and Meek [38] and Focks *et al*. [39] reported that soil moisture is a major factor affecting the abundance of some species. However, measurements of soil moisture were not included in this study.

We did not found any significant association with temperature; mosquito density and malaria incidence in our study. Certainly, temperature is directly affecting mosquito breeding, survival, and behavior and also malaria transmission [40–43]. We were unable to detect a significant relationship with this factor, because the temperature ranges in this region are always suitable for mosquito breeding and development. Moreover, statistical significance alone does not always unclouded the complex biological dynamics of mosquito and temperature.

Though, rainfall is the major key factor to enhance the malaria transmission in several countries [44–46]. However, it was negatively correlated with malaria cases in India [47]. We found moderate relation (R = 0.573, P = 0.052) between the number of malaria cases and rainfall in Bangladesh which was supported by Gupta [48]. Haque *et al*. [2] investigated the relationship between climatic parameters and malaria cases over the last 20 years in the malaria endemic district of Chittagong hill tracts of Bangladesh and showed insignificant relation. But, Wiwanitkit [49] reported that malaria cases were positively associated with rainfall in Thailand. Briet *et al*. [50] showed that malaria cases increased with lower rainfall and that the region with the highest rainfall had the least malaria. Malaria incidence and relative humidity were positively associated when not considering the effect of multiple factors. However, no association was observed in our study when computed combined effect of multiple factors. Bhattacharya *et al*. [47] accounted humidity levels between 55 and 80% were appropriate for both *P. falciparum* and *P. vivax* and this range of humidity are present throughout the year in Bangladesh. It was also reported that the malaria risk at 80% humidity was double as that of 60% [42, 51].

Based on the observation of *Anopheles* monthly distribution and malaria prevalence, *An. baimaii*, *An. willmori*, *An. vagus*, and *An. subpictus* seem to potential vector of malaria in the study area. This hypothesis was supported by their anthropophilic and opportunistic feeding behavior [52] and *Plasmodium* infection rate [1, 9].

## Conclusions

The study demonstrates that the incidences of malaria all year round in Kumari, Bangladesh, due to the favorable environmental conditions. Therefore, integrated vector management system covering year round should be adopted to reduce of malaria morbidity and mortality in Bangladesh. Additionally, basic and applied research on the ecological, social and economic determinants of the disease is required to promote the regular assessment of a country’s malaria situation. Like other studies, we did not find significant relationships of rainfall and temperature with all anophelines; even so, this may be due to the fact that other studies used diverse methodologies in different regions of the globe, where the vector ecology is pretty different. Multiple environmental factors are responsible for mosquito breeding and malaria infection in the Bangladesh. This study recommends vector ecologists to cautiously consider the complex nature of the relationship between malaria vectors and climate variables. Detailed studies of vector bionomics, continuous monitoring and malaria transmission dynamics is essential for predicting outbreaks of disease and, if necessary, control of pest mosquitoes in Bangladesh.
